# Exploring the social and behavioral barriers to hypertension self-care among indonesian adults: a qualitative study based on the theory of planned behavior

**DOI:** 10.1186/s12889-026-27470-6

**Published:** 2026-05-11

**Authors:** Andi Mayasari Usman, Cecep Eli Kosasih, Iqbal Pramukti, Yulia Sofiatin, Rian Adi Pamungkas

**Affiliations:** 1 Medicine Department, Faculty of Medicine, Universitas Padjadjaran, Bandung, Indonesia; 2 Nursing Department, Faculty of Health Science, Universitas Nasional, South Jakarta, Indonesia; 3 Nursing Department, Faculty of Nursing, Universitas Padjadjaran, Bandung, Indonesia; 4 Department of Epidemiology, Faculty of Medicine, Universitas Padjadjaran, Bandung, Indonesia; 5 Nursing Department, Faculty of Health Sciences, Esa Unggul University, West Jakarta, Indonesia

**Keywords:** Hypertension, Self-care, Social-behavior barriers, Family support, Theory of Planned Behavior, Health behavior

## Abstract

**Background:**

Sustaining hypertension self-care practices presents ongoing challenges, particularly in socially complex and resource-limited settings. Although the Theory of Planned Behavior (TPB) is widely used in health behavior research, its applicability in social and behavioral contexts remains underexplored. This study aimed to extend the TPB by identifying social-behavioral constructs that influence self-care behavior in hypertension.

**Methods:**

A qualitative descriptive study was conducted in Jatinangor District, West Java, Indonesia. Adult participants included patients with uncontrolled hypertension, family members, community health workers (cadres), and health workers. Twenty-four key informants who fulfilled these criteria were recruited for the study. Data were collected through semi-structured interviews and analysed using thematic content analysis guided by TPB constructs. Ethical approval was obtained from the Padjadjaran University, and informed consent was obtained from all participants.

**Results:**

Five themes were identified as barriers to sustainable self-care: 1) lack of information and misperception (attitude), 2) low perception of susceptibility and severity (perceived behavioral control), 3) lack of family support and insufficient workforce (subjective norms), 4) conventional customs of gathering and culture of eating habits (subjective norms), and 5) doubt about the benefits and efficacy of hypertension treatment (attitude). These themes illustrate how social and behavioral contexts, including family interactions and daily habits, shape TPB constructs.

**Conclusions:**

This study contributes to the theoretical development of TPB by embedding social-behavioral constructs that influence hypertension self-care in low-resource settings. These findings highlight the need for socially responsive and family-oriented strategies to strengthen self-care for hypertension. These insights are relevant for designing sustainable intervention in low- and middle-income countries (LMICs), particularly in Southeast Asia.

**Supplementary Information:**

The online version contains supplementary material available at 10.1186/s12889-026-27470-6.

## Introduction

Hypertension is a primary global health concern and poses a significant burden in terms of morbidity and mortality [1]. The World Health Organization (WHO) in 2023 estimated that more than 1.28 billion adults were living with hypertension, especially in lower-middle-income countries with limited accessibility to health services [2]. In Indonesia, the prevalence among adults has reached 30.8% [2], underscoring the urgent need for effective management strategies.

Self-care is a cornerstone of hypertension management, including medication adherence, dietary modification, and physical activity, and routine blood pressure monitoring. These behaviors are proven to reduce complications such as stroke, heart failure, and kidney disease [3, 4]. However, sustaining long-term engagement remains a challenge. Social relationships, family dynamics, and behavioral patterns strongly influence perceptions of risk and control. Despite their significance, social-behavioral determinants are often overlooked in conventional hypertension interventions, which tend to emphasize biomedical approaches rather than context-specific strategies [3–5].

Evidence from various settings demonstrates barriers to sustained self-care. In the United States, patients with hypertension have reported difficulties in sustaining self-care behaviors due to social and structural barriers such as limited motivation for physical activity, financial obstacles to medication access, and inadequate engagement with healthcare services, highlighting the role of patient-provider communication in adherence [6]. Another study revealed that a poor understanding of hypertension, limited access to sustainable treatment, and ineffective communication with health providers are critical obstacles [7].

These challenges are rooted in behavioral routines and social norms that influence health-seeking behaviors rather than cultural customs alone [8]. Furthermore, dietary practices, especially the high consumption of sodium rich traditional foods, continue to hinder effective blood pressure control [9, 10]. Despite government initiatives, including “Pos Pembinaan Terpadu Penyakit Tidak Menular” (Posbindu PTM), a community-based integrated post aimed at supporting the early detection and management of non-communicable diseases such as hypertension, the implementation of these programs remains inconsistent. Key challenges include shortages of trained health workers, limited continuity of follow-up, and suboptimal patient engagement, particularly in semi-urban and resource-limited settings [11].

The Theory of Planned Behavior (TPB) has been widely employed to explain and predict health-related behaviors through three central constructs: attitudes, subjective norms, and perceived behavioral control [12]. Attitude refers to an individual’s positive or negative evaluation of performing a particular behavior; subjective norms reflect perceived social pressure from significant others to perform or not perform the behavior; and perceived behavioral control represents an individual’s perception of their ability to execute the behavior despite potential barriers. Empirical studies applying TPB in the context of chronic disease management have primarily focused on the individual-level determinants of health behavior. For instance, favorable attitudes toward treatment benefits and higher perceived behavioral control have been associated with better adherence to hypertension self-care behaviors, including medication adherence, dietary modification, and routine blood pressure monitoring [12, 13]. In addition, subjective norms particularly encouragement and expectations from family members and healthcare providers have been shown to strengthen individuals’ intentions to engage in recommended self-care practices [13].

Although TPB is a robust and widely used model in health behavior research, its application has primarily focused on individual-level determinants, with limited attention paid to how cultural and familial contexts may shape these constructs. In collectivist societies such as Indonesia, family dynamics, communal norms, and culturally embedded practices, particularly those related to diet and daily routines can substantially influence health-related decision-making and self-care behaviors [14].

**Fig. 1:**
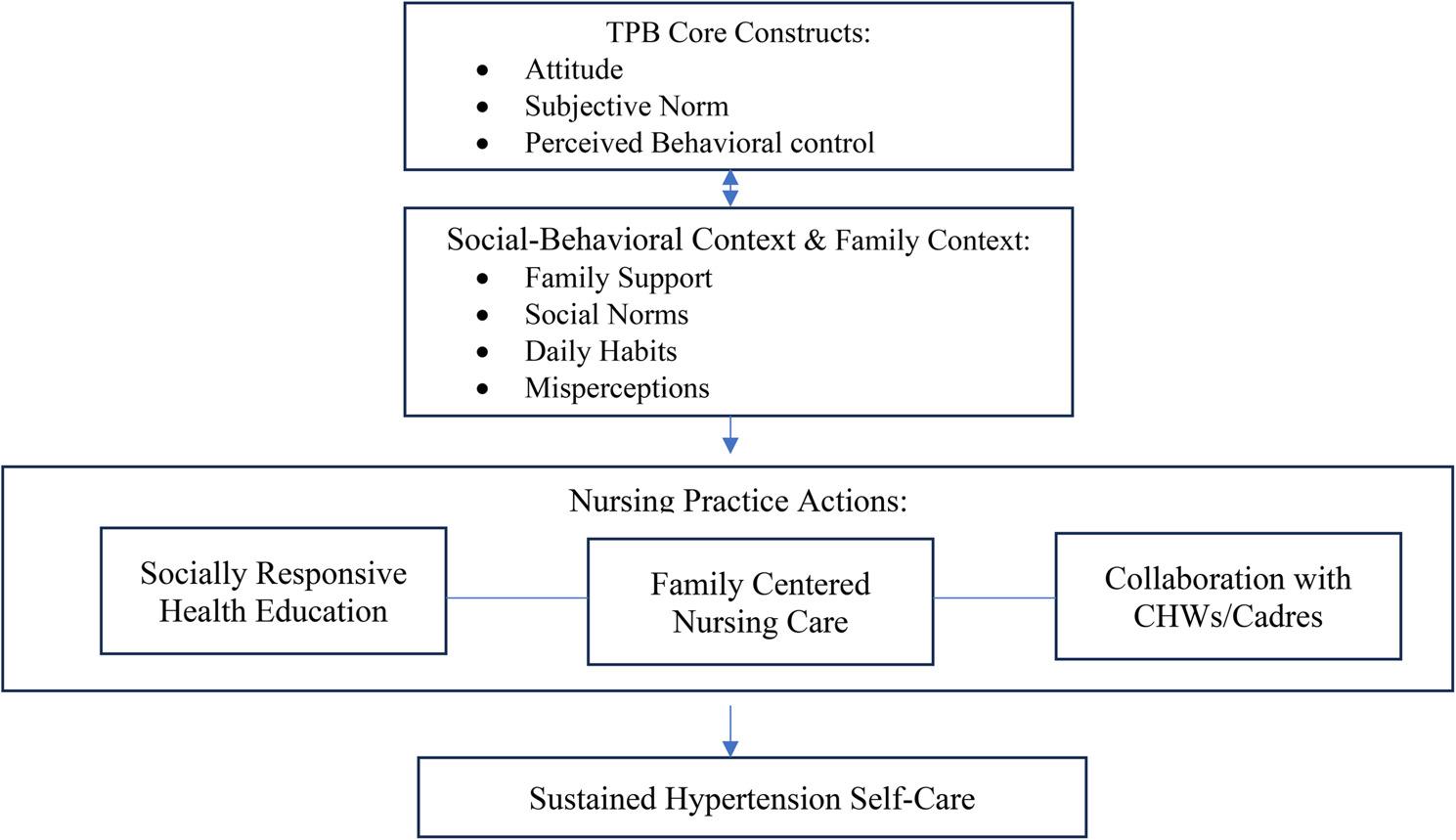
Conceptual framework illustrating the application of the Theory of Planned Behavior (TPB) to hypertension self-care, incorporating social-behavioral and family contexts and their implications for community nursing practice

Therefore, this qualitative study aimed to explore social and behavioral barriers to sustaining hypertension self-care by applying the Theory of Planned Behavior as an analytic framework, with particular attention to the influence of family and sociocultural contexts in a low-resource Indonesian setting. Figure 1 presents the conceptual framework of this study, illustrating the contextual application of TPB and its relevance to community nursing practice.

## Methods

### Study design and setting

A descriptive qualitative study was conducted using in-depth interviews. This study was conducted at the Jatinangor Health Center, Sumedang, West Java, Indonesia. Jatinangor is a semi-urban subdistrict with the third-highest population density in the region, representing a typical Indonesian community experiencing rapid demographic shifts and increasing non-communicable disease burdens. The site was purposively selected because of its diverse socioeconomic composition, healthcare access disparities, and growing epidemiological burden of hypertension. These characteristics mirror the conditions in many semi-urban areas across Indonesia, making Jatinangor a representative setting for exploring social behavior and systemic barriers to sustainable hypertension self-care. Local public health reports also highlight the high prevalence of uncontrolled hypertension in this area, further supporting its relevance as a study location.

### Population and samples

We included individuals living in the Jatinangor area who were directly involved in treatment and had experiences and insights related to self-care management. The key informants included patients with hypertension, families, community health workers, and nurses.

The sample for this study consisted of 24 key informants who were intentionally selected based on specific inclusion criteria and invited to participate in the interviews (see Table 1). The justification for the sample size of 24 informants was determined by the depth and richness of data rather than statistical representativeness. Data collection continued until thematic saturation was achieved, which was defined as the point at which no new themes or insights emerged from successive interviews across informant groups. Saturation was achieved with 24 participants, which provided sufficient variation and depth to address the study objectives. Empirical evidence from a systematic review by Hennink and Kaiser indicates that saturation in interview-based qualitative studies is commonly achieved within 9–17 interviews, and may extend to up to 24 interviews when studies aim to capture meaning saturation across multiple informant groups [15]. Given the inclusion of four distinct informant groups, a sample of 24 participants was methodologically appropriate and sufficient to address the study objectives. 

**Table 1:** Overview of informants and Inclusion Criteria

Informants	Number of Participant	Gender (F/M)	Age Range	Inclusion Criteria
Patients with uncontrolled hypertension	8	7/1	35–55	• The minimum age is 35 years. • Individuals must have been diagnosed with hypertension for a minimum of one year.
Family members	8	4/4	27–57	• Caring for Patients with Hypertension • Providing care for hypertension patients for a minimum of one year.
Health Community workers (Cadre)	5	5/0	39–60	• Support the management of blood pressure and salt preferences in the community. • Have at least one year of experience in hypertension care.
Person in charge of PTM (Non-Communicable Diseases) and PROLANIS (Chronic Disease Management) programs	3	3/0	39–47	• Participate in the care of patients with hypertension at health centers. • Involved in a hypertension management program for at least one year.
Total	24			

The first group included hypertensive patients with uncontrolled blood pressure who had lived in Jatinangor for at least one year and were willing to participate in the study. The second group was comprised of eight family members who were directly involved in caring for the hypertensive patients and lived with them. The third group consisted of five community health workers who managed patients with hypertension. The fourth group included nurses who participated in the hypertensive patient care program in the Jatinangor area and had one year of experience caring for hypertensive patients in the community. Both cadres and nurses played direct roles in health checks, blood pressure monitoring, home visits, health education, and follow-up care.

### Instrumentation

The semi-structured interview guide was developed based on an extensive review of the literature on hypertension self-care management, salt preference, and the Theory of Planned Behavior (TPB). The interview questions were explicitly mapped to the three core constructs of the Theory of Planned Behavior: attitude, subjective norm and perceived behavioral control to ensure comprehensive coverage of participants’ beliefs, social influences, and perceived barriers and facilitators related to hypertension self-care, while also incorporating locally relevant social and family contexts influencing daily self-care practice.

Content validation was conducted through an expert panel consisting of a community nursing expert, a medical doctor, a nutritionist, and a medical anthropologist. The experts evaluated the interview guide for relevance, clarity, cultural appropriateness, and conceptual alignment with TPB constructs. The evaluation criteria included the appropriateness of each question in capturing the intended TPB construct, clarity of wording, cultural sensitivity, redundancy, and logical sequencing of the questions. Items were reviewed qualitatively and refined based on expert judgment and consensus rather than relying on quantitative scoring. No formal statistical validation (e.g., Content Validity Index) was performed, as the study adopted a qualitative design emphasizing expert judgment, pilot testing, and contextual relevance.

Prior to formal data collection, the interview guide was pilot tested with five hypertensive patients from a community outside the study site to assess comprehension, cultural relevance, and the flow of questions. Minor revisions were made based on participant feedback to clarify the terminology related to dietary practices and family involvement in hypertension self-care. During the early phase of data collection, the interview guide was applied flexibly, allowing for minor iterative refinements when participants indicated ambiguity or misunderstanding. This approach ensured methodological rigor, while maintaining responsiveness to participants’ experiences and sociocultural contexts. The final English version of the semi-structured interview guide for each informant group is provided in Supplementary File 1.

### Data collection procedure

In-depth interviews were conducted using a semi-structured format with open-ended questions to explore experiences and barriers related to hypertension self-care management through the lens of the Theory of Planned Behavior (TPB). Interviews with patients with uncontrolled hypertension, their family members, and community health workers were conducted in the participants’ homes, while interviews with nurses were conducted at the health center. Each interview session lasted for approximately 60 min.

The interviews were conducted in Indonesian. All interviews were audio-recorded with participants’ consent and transcribed verbatim in Indonesian. The transcripts were subsequently translated into English for analysis and reporting purposes. To ensure translation accuracy and conceptual equivalence, the translated transcripts were reviewed by the research team and any discrepancies were discussed and resolved through consensus.

The interview guide was structured according to four key informant groups. Interviews with patients explored perceptions of hypertension, attitudes toward self-care and dietary salt intake, subjective norms, perceived behavioral control, and barriers to sustained self-care. Family member interviews focused on caregiving roles, family attitudes, and challenges in supporting the management of hypertension. Interviews with community health workers addressed their roles in assisting blood pressure control, dietary counseling, and barriers encountered in community-based care. Interviews with nurses explored the systemic and programmatic challenges of implementing hypertension management and salt reduction initiatives.

Data collection was conducted by the principal researcher with the assistance of a trained research assistant. The principal researcher is a nursing academic with expertise in chronic disease management, particularly hypertension self-care, and experience in applying behavioral theories such as the Theory of Planned Behavior (TPB) in both qualitative and intervention-based research. The research assistant is a trained health professional with experience in community-based programs and qualitative data collection. Prior to data collection, the research assistant received training in qualitative interviewing techniques, the TPB framework, and hypertension management to ensure consistency and depth in the data collection process. The interview guide had been previously validated and pilot-tested, as described in the Instrumentation section.

### Data analysis

The data were transcribed and translated from the audio recordings. A deductive content analysis approach guided by the theoretical constructs of the TPB (attitudes, subjective norms, and perceived behavioral control) was employed. The data analysis process consisted of several steps. First, we read the transcript repeatedly to obtain immersion data. The meaningful information related to the TPB model and the aims of this study were the initial coding. Following the data collection, a data reduction process was conducted to identify the initial themes and subthemes aligned with the theoretical framework. Content analysis was then used to systematically code and categorize the data, allowing for the identification, analysis, and reporting of themes within the local social-behavior context. A process of constant comparison across transcripts from different informant groups of patients, family members, community health workers, and nurses was used to refine themes and identify similarities and variations in perspectives. Thematic saturation was considered to be achieved when no new codes or themes emerged from successive interviews, and additional data did not generate new conceptual insights but reinforced existing categories. Saturation was assessed across informant groups rather than within a single category to ensure consistent patterns across perspectives. The final stage involved organizing these themes into categories and subcategories, reflecting the perspectives of each key informant group such as patients, families, volunteers and nurses who are responsible for NCD.

To enhance trustworthiness, two researchers independently coded a set of transcripts and compared their results. Inconsistencies in the information were discussed until a consensus was achieved. Triangulation was applied by comparing perspectives across the four different informant groups to validate recurring patterns and to highlight contextual variations. In addition, member checking was conducted with the selected participants to confirm that the interpretations accurately represented their experiences. These strategies collectively enhanced the credibility, dependability, and conformability of the findings. The principal had experience in community health and hypertension management, which may have influenced the interpretation of participants’ narratives. Reflexivity was supported through regular discussions within the research team, iterative comparison of interpretations, and consensus building during data analysis to minimize potential bias.

### Theoretical framework

This study applied the Theory of Planned Behavior (TPB) to explore the social behavioral barriers to hypertension self-care. The TPB’s core constructs: attitudes, subjective norms, and perceived behavioral control, guided the analysis of how patients and families manage self-care daily. Family involvement, particularly among cohabiting relatives, significantly shaped patients’ intentions and behaviors and was influenced by cultural norms on diet, health beliefs, and shared responsibilities.

Input from nurses and community health workers (cadres) illuminated the systemic barriers to program implementation. Nurses reported challenges such as workforce shortages and limited community participation, while cadres encountered cultural resistance and logistical constraints in delivering community-level support.

Integrating insights from all stakeholder groups allowed for contextual refinement of the TPB framework and socially embedded factors influencing self-care. These findings inform the development of community-based interventions that are both socially responsive and feasible in resource-limited, semi-urban settings, such as Jatinangor.

## Results

### Social-behavior barriers to hypertension self-care practice

Five themes were identified as barriers to sustainable self-care: These themes illustrate how social-behavior and psychosocial contexts interact with the constructs of the TPB.

### Theme 1: lack of information and the misperception (Attitude)

Most patients received information about self-care management for hypertension from healthcare workers and nurses at the health centers. However, they still lack a comprehensive understanding of the information provided, which is often delivered in a one-way manner. Patients believe that high salt intake is the primary cause of high blood pressure; however they are unaware of which foods are high in salt or how to prepare food to reduce its salt content. Their understanding of hypertension is limited to its definition, and they believe that high blood pressure could potentially lead to a stroke. They were not informed about the other possible consequences of uncontrolled blood pressure. Additionally, they mistakenly believe that blood-boosting medications are intended to increase blood pressure, rather than to understand their true purpose. Other factors contributing to elevated blood pressure are also poorly understood. Many patients believe that healthy foods are synonymous with expensive foods.

The family members who cared for patients with hypertension conveyed their challenges in managing the care plan. They were not fully aware of how to effectively care for individuals with hypertension or prepare appropriate home-cooked meals. However, one family successfully managed their diet as they had previously dealt with hypertension. In the in-depth interviews conducted with community health workers, the coordinator observed an opportunity to improve the community’s understanding of the factors that lead to uncontrolled blood pressure. Many believe that medication could lower blood pressure, neglecting the importance of a healthy lifestyle. Health center nurses reported challenges in educating patients owing to a shortage of healthcare workers. Consequently, the education provided is suboptimal.

“*We cannot eat healthy food often because we have to drink milk and fruit. It is too expensive.”* (P7, 35-year-old female).

“*I struggle to manage my blood pressure because I do not understand how to prepare healthy meals or what affordable healthy foods I can consume.”* (P8, 52-year-old female).

“*When the health center provides me with blood-boosting medicine*,* I refrain from taking it because my blood pressure would increase.”* (P3, 40-year-old female).

“*Reducing my salt consumption should help lower my blood pressure. I now try to cook with less salt since I previously had hypertension*,* but it is currently under control*.” (KE1, 56-year-old mother).

“*People here believe that food lacks flavor without salt*,* masako*,* or sasa. Thus*,* these three ingredients are commonly used in dishes.”* (KA3, 39-year-old female).

### Theme 2: low perception of susceptibility and severity (Perceived Behavioral Control)

Lack of awareness of the risk and severity of the illness leads to non-adherence to the main programs, putting their health at risk and increasing disease complications. Five informants reported that they did not consistently take their hypertension medication or follow care management guidelines for various reasons, including financial limitations, lack of understanding of the consequences of non-adherence, and ingrained environmental habits.

In addition to the structural and informational barriers, several participants expressed psychological avoidance of routine health examinations. Rather than logistical constraints, this avoidance stemmed from a fear of being diagnosed with additional illnesses, which they believed could lead to excessive worry and emotional distress, potentially exacerbating their blood pressure levels.

“*I take medication for hypertension. If my blood pressure increased*,,* I took amlodipine.”* (P1, 48-year-old female).

“*I rarely visit the integrated health post or health center because I have no complaints.”* (P5, 55-year-old male).

*“I dedicated myself to a consistent workout routine*,* pushing my limits at the exercise*,* but my blood pressure remained high. Eventually*,* I became lazy about working. Whenever I had a headache*,* I choose to rely on medication instead of directly confronting the underlying problem. I enjoy foods such as salted fish and do not have any specific dietary restrictions. If my blood pressure increases*,* it is easy to visit the pharmacy to buy amlodipine.”* (P2, 36-year-old female).

“*I am confused about my rising blood pressure despite reducing my salt intake. My mother also cooks for less salt. It seems that the increase is due to stress rather than food*”. (P6, 35-year-old male).

*“I am scared to go for a check-up. What if they find something bad? I will keep thinking about it*,* and that can make my blood pressure go up again.”* (P3, 40-year-old female).

*“Better not to know. If I find out I have another disease*,* it will just make me overthink and get more anxious. It is easier not to worry*.” (P8, 52-year-old female).

*“Some people say they do not want to get checked because they are afraid. If they know they are sick*,* they say it will make them panic or stress too much*.” (KA1, 58-year-old female).

*“They often say*,* ‘If I do not know*,* I feel fine. If I know*,* I might just get worse from overthinking.’ So they avoid going to Posbindu or the health center*.” (KA3, 39-year-old female).

### Theme 3: lack of family support and insufficient workforce (Subjective Norms)

Family support is a particular element in managing blood pressure because family members live with patients and influence their daily activities. Family habits and dynamics can significantly shape an individual’s mindset and lifestyle. However, certain family behaviors, such as cooking salty foods or engaging in social gatherings that involve eating, can pose challenges for young individuals with hypertension while maintaining a healthy diet. Additionally, the limited number of healthcare workers can hinder the effectiveness of health programs, making it difficult to reach all individuals within a health center’s service area. Seven patients reported difficulties accessing health services, particularly at health centers. Distance, cost, and lack of available facilities are significant obstacles to effective hypertension management.

“*I always participate in botram*,* bringing food from home. Sometimes we eat salted fish or fried tempeh. Eating together is delicious*,* and we can chat while enjoying the meal*,* so we often do this.”* (P1, 48-year-old female).

“*I prepare the daily meals for my family*,* who prefer salty foods*,* sometimes accompanied by fries. My husband and children eat what I cook.”* (P2, 36-year-old female).

“*I seldom visit the health center for check-ups because it is far away. I prefer to spend money on food instead.”* (P7, 35-year-old female).

“*Going to the health center requires a long trip*,* and online transportation is expensive*,* so we rarely visit.”* (P3, 40-year-old female).

### Theme 4: conventional customs of gathering and the culture of eating habits (Subjective Norm) 

Local Sundanese culture, particularly the tradition of botram (communal eating), significantly influences blood pressure management and salt preferences. It is not the act of sharing a meal that directly leads to increased blood pressure but rather the types of food typically served at botram gatherings. These foods, such as salted fish, fatty dishes, and crackers. Are high in salt. As a result, this eating habit can make it challenging for individuals to manage their blood pressure, even though they understand the importance of maintaining a low-salt diet. The foods served are popular among Sundanese people, and the communal nature of these gatherings often increases their appetites.

*“I often visit Botram almost every day; they serve home-cooked food like salted fish and fried tempeh. This might be the reason for my uncontrolled blood pressure”. (*P7, 35-year-old female*).*

*“It can be challenging to reduce salt*,* as the menu at Botram often features salted fish and crackers.” (*P3, 40-year-old femal).

“*In my family*,* I enjoy eating salted fish*,* which is why I have hypertension. My mother also has hypertension because she enjoys salted fish*,* shrimp paste*,* chili sauce*,* and crackers.”* (P7, 35-year-old female).

“*I struggle to manage my eating habits. We often come together here because if we stay home all the time*,* our headaches and blood pressure tend to increase. In the evenings*,* we chat with the neighbors while enjoying snacks*,* such as meatballs and other treats*,* to relieve our stress.”* (P3, 40-year-old female).

### Theme 5: doubt about benefits and efficacy of hypertension treatment (Attitude)

Understanding the risks and severity of hypertension is crucial because it directly influences patients’ motivation to manage their condition effectively. The more patients are unaware that they are at risk of uncontrolled blood pressure and potentially severe complications associated with hypertension, the more non-compliant they are with their treatment and adopt unhealthy lifestyles. Many patients mistakenly believe that they only need to take medication when they experience symptoms, thinking that it will lower their blood pressure without considering necessary changes in their diet and lifestyle. Even if they engage in healthy behaviors, such as exercising regularly and reducing salt intake, they may still find that their blood pressure remains high.

“*I do not experience any of the symptoms that often accompany high blood pressure*,* which leads me to believe that there is no real problem*,* even though my blood pressure is elevated.”* (P5, 55-year-old male).

*“I do not take medication regularly because it tends to give me* a headache.” (P1, 48-year-old female).

*“I dedicated myself to a consistent workout routine*,* pushing my limits at the exercise*,* but my blood pressure remained high. Eventually*,* I became lazy about working out. Whenever I had a headache*,* I choose to rely on medication instead of directly confronting the underlying problem. I enjoy foods such as salted fish and do not have any specific dietary restrictions. If my blood pressure increases*,* it is easy to visit the pharmacy to buy amlodipine.”* (P2, 36-year-old female).

“*My family and I enjoy salty foods. Sometimes*,* after adding salt to our meals*,* I sprinkle more Sasa or Masako. When I have a headache*,* I suspect it is due to high blood pressure*,* so I usually go to sleep and take some medicine. I am wondering what I can do about this habit.”* (P3, 40-year-old female).

**Table:** 

Categories	Thema	Sub-Theme
Attitude	Lack of information and the misperception	1. Misunderstandings about hypertension and its management 2. Lack of knowledge about proper blood pressure control 3. Limited access to educational media and health services 4. Ineffective one-way communication in health education
Perceived Behavioral Control	Low perception of susceptibility and severity	1. Uncertainty about the benefits and effectiveness of treatment 2. The misconception that hypertension is not a severe disease. 3. The widespread belief that treatment is only administered in response to visible symptoms 4. Avoidance due to fear of diagnosis and emotional distress 5. Emotional burden and fear of receiving bad news Belief that worry can worsen hypertension
Subjective Norm	Lack of family support and insufficient workforce	1. Lack of social support in managing blood pressure 2. Family habits related to diet 3. High workload and job demands Lack of self-confidence in managing hypertension
Subjective Norm	Conventional customs on gathering and the culture of eating habits	1. Cultural preferences for high-salt foods 2. Social pressures on communal eating habits 3. Family traditions that conflict with dietary restrictions 4. Lack of awareness about the impact of traditional foods on blood pressure
Attitude	Doubt about the benefits and efficacy of hypertension treatment	1. Concerns about potential long-term side effects from treatment. 2. The belief is that medication alone is sufficient. 5. Misinformation regarding reliance on antihypertensive medications.

## Discussion

This study explored the social and behavioral barriers to sustainable self-care, guided by the Theory of Planned Behaviour (TPB). The findings emphasize how social relationships, behavioral routines, and systemic constraints interact with attitude, subjective norms, and perceived behavioural control in shaping self-care behaviors. In this theory, intention refers to a person’s commitment to engaging in specific behaviours as a bridge between psychosocial and cognitive factors, and actual behaviour. Individuals are more likely to successfully implement these behaviours than those without such intentions [13]. Using the TPB theory approach in qualitative research, five main themes were found in this study: (1) lack of information and the misperception, (2) low perception of susceptibility and severity, (3) lack of family support and insufficient workforce, (4) conventional custom on gathering and culture of eating habits, and (5) doubt about the benefits and efficacy of hypertension treatment. While this study focused on salt preference as a key behavioral target, other self-care behaviors such as physical activity, medication adherence, and stress management also emerged during interviews, particularly within the perceived behavioral control construct, highlighting the broader behavioral context of hypertension self-care. These findings further underscore the role of cognitive and perceptual factors, particularly patients’ understanding and beliefs about hypertension, in shaping health behaviors.

Misperceptions and doubts about treatment efficacy reflect negative attitude that reduce patients’ intentions and adherence, consistent with prior studies showing that limited health literacy is associated with poor hypertension control [16]. A study conducted by Coughlin et al. indicated that a lack of information and misunderstandings about disease management lead to delayed diagnosis and treatment. This delay can negatively affect health outcomes and increase the risk of complications [17]. Other studies also suggest that misconceptions about disease management can result in poor self-management skills, worse health outcomes, and higher reliance on health services [18]. A study conducted by Severin et al. found that misinformation and false beliefs can drive patients to pursue alternative treatments that lack scientific evidence, potentially resulting in catastrophic health consequences. In addition, our study found that behavioral intention to change is formed when there is an integration of attitude, subjective norms, and perceived behavioral control. Addressing these gaps in understanding is vital for safeguarding patient well-being and improving overall health outcomes [19].

One of the most critical challenges in self-care for patients with hypertension is their insufficient awareness of the risks and severity of their condition. This knowledge gap often leaves patients either uninformed or has a weak understanding of the potential dangers and severe consequences associated with the disease [20]. Consequently, they may experience delays in diagnosis, fail to adhere to treatment protocols, and face an increased risk of serious complications. Research conducted by Tan et al. revealed that when patients are unaware of the dangers posed by hypertension, they are significantly less likely to take proactive steps toward prevention and treatment adherence [12]. Moreover, other studies have demonstrated that individuals lacking awareness of hypertension’s risks, according to the Health Belief Model (HBM), exhibit notably lower compliance with both medical recommendations and necessary lifestyle changes [21], and findings from Jee-Seon Shim, et al. indicate that those who underestimate hypertension risks are less inclined to follow medical advice and maintain dietary adjustments, such as a low-salt diet [22].

The third theme identified in this study was the lack of family support and the insufficiency of mentors in managing blood pressure control. Family support includes emotional, informational, and practical assistance from family members, which is essential for helping patients with hypertension manage self-care. Within the TPB framework, subjective norms, such as family influence are pivotal in shaping patient intentions and behaviors. This was consistent with prior research, which found that families often lacked the knowledge and skills to support dietary modifications and medication adherence, thus undermining patients’ ability to manage their condition effectively [23]. Family members are responsible for educating and assisting patients in disease management and behavioral changes [24]. Families with a clear understanding of a patient’s health can provide better support for enhancing the patient’s overall health.

In addition to family support, peer mentorship is an essential element in empowering patients to control their hypertension. These mentors have triumphed over their battles with high blood pressure and have served as inspiring role models, educators, and guides within the community. Patients who witness the success stories of those who have effectively managed their condition often find renewed confidence in and commitment to their treatment plans. Kalantzi et al. revealed that many patients resist lifestyle changes due to uncertainty about their ability to succeed [25]. Exposure to the experiences of those who have successfully navigated similar challenges can spark a stronger motivation to pursue health goals and actively adopt the recommended wellness strategies. Furthermore, studies have consistently demonstrated that peer mentors play a critical role in alleviating the pervasive fear, anxiety, and stress associated with hypertension. Many patients feel isolated during their struggles. However, these mentors provide vital emotional and social support, sharing proven strategies and personal success stories that significantly empower patients to improve their health outcomes [22].

The fourth theme in this study was the conventional customs of gathering and communal eating habits. This theme discusses eating habits and social interactions related to food consumption patterns, including salt and fat intake levels, as well as the cultural significance of social gatherings. Culture significantly influences health by affecting adherence to hypertension management diets through social habits and culinary traditions. Embracing a culture-based approach for self-care management is more effective than imposing outright restrictions on certain foods. We can enhance patients’ long-term commitment to dietary guidelines by fostering a connection with cultural practices. This finding is in line with a study confirming that food consumption is deeply embedded within social and cultural traditions, where eating together reinforces social bonds but also normalizes a high intake of salt and fatty foods [26–28].

Another study showed that lifestyle modifications aligned with cultural adjustments in diet, physical activity, and stress management have significantly boosted patients’ adherence to dietary recommendations for hypertension [29]. A recent study by Altawili et al. demonstrated that culturally adapted diet education is more effective than generic diet education in improving the understanding of local eating habits and cultural practices [30]. This approach has proven particularly beneficial for dietary interventions aimed at patients with hypertension.

Research in the United States shows a troubling trend among Filipino immigrants: a transition to a Western diet high in fats and sugars linked to rising blood pressure and increased hypertension rates. Alarmingly, their traditional diet typically includes an average sodium intake of 12 g/day, which is eight times the American Heart Association’s recommended limit. Excessive sodium consumption significantly increased the risk of hypertension. This situation shows the critical impact of cultural shifts and dietary modifications can have on public health, leading to a troubling rise in hypertension within this population [31, 32]. A recent study revealed that the Mediterranean diet, which is rich in fruits, vegetables, and healthy fats, is closely associated with a significantly lower risk of cardiovascular disease and hypertension. This robust evidence underscores the crucial role of dietary choices play in shaping an individual’s health [33]. By understanding the social and behavioral contexts within local communities, nurses can deliver more effective care and reduce the risk of miscommunication.

The fifth theme in this study addressed uncertainties regarding the advantages and effectiveness of hypertension treatment. These uncertainties often manifest as scepticism or disbelief in the benefits and efficacy of treatment, stemming from various factors such as personal experiences, misinformation, and a limited understanding of hypertension management. Patients who doubt the benefits of treatment show non-compliance with medication and a lack of motivation to engage in health programs including health checks, exercise, and dietary changes. Similarly, a study on low-resources confirmed that skepticism in treatment often arises when cultural explanations of illness conflict with medical advice, leading to treatment delays or inconsistent adherence [34–36].

Another study indicated that health interventions, particularly those involving education and support combined with a comprehensive approach, are highly effective in dispelling doubts about the value of blood pressure monitoring and fostering greater patient engagement [37]. A previous study mentioned that delivering accurate and current information is vital for transforming health behaviours. It not only alleviates uncertainties, but also builds trust in treatment [38]. The theory of planned behavior (TPB) interpreted the uncertain attitudes toward treatment with low behavioural intention, and limited perceived control was significantly associated with poor adherence. Therefore, the present findings reinforce the need for comprehensive, socially responsive, and family inclusive strategies in hypertension self-care programmes.

These findings reinforce the relevance of integrating social behavior determinants within the TPB framework to better understand self-care behaviours in hypertensive populations. While traditional TPB applications emphasize individual cognition, this study extends the Theory of Planned Behavior by embedding social and behavioral dimensions, specifically social norms, family routines, and habitual behaviors, into the interpretation of attitude, subjective norm, and perceived behavioral control. These findings underscore the need for socially responsive and behavior-oriented interventions that engage family, peers, and community systems to sustain self-care for hypertension.

### Implication for practice

This study highlights critical considerations for community nursing practice in culturally diverse and resource-limited contexts. Nurses should prioritize socially responsive health promotion strategies that align with local beliefs and dietary habits to enhance patient engagement and promote reduced salt consumption and consistent blood pressure monitoring. Tailored education may foster greater patient engagement in and adherence to blood pressure monitoring.

Family centered interventions should be prioritized, given the prominent role of families in shaping on dietary habits, medication adherence, and care-seeking. Nursing practices should integrate family-based sessions, home visits, and group discussions into existing community health programs to enhance continuous support for patients with hypertension.

At the community level, partnerships with health workers (cadres) are vital for expanding outreach and bridging gaps in service delivery. Strengthening cadres through training, supervision, and accessible educational tools can improve program sustainability. Finally, at the policy and professional development level, the TPB should be incorporated into continuing education to enhance nurses competencies in addressing attitudes, norms, and perceived control. Multidisciplinary collaboration for socially responsive and community driven care models is essential to sustain hypertension self-care initiatives.

### Strengths and limitations

This study used a qualitative design that allows for an in-depth exploration of social and behavioral barriers to hypertension self-care from multiple perspectives, including patients, family members, community health workers, and nurses. The use of the Theory as an analytic framework provided a systematic lens to interpret how attitudes, subjective norms, and perceived behavioral control operate within social and family contexts. In addition, data triangulation across different informant groups and rigorous analytical procedures enhanced the credibility of the findings. Nevertheless, several limitations should be acknowledged. The study was conducted in a single semi-urban setting, which may limit the transferability of the findings to other contexts with different cultural or healthcare characteristics. As with all qualitative research, the findings are based on participants’ self-reported experiences and may be subject to recall or social desirability bias. The study also did not explicitly examine the role of educational level, which may influence health literacy and self-care behavior. Future research should consider incorporating educational background as an important determinant in understanding hypertension self-care. Despite these limitations, methodological rigor was maintained through careful study design, data triangulation, and reflexive analysis.

## Conclusions

This study offers a nuanced and contextually grounded understanding of the social and behavioural barriers impeding the sustainability of self-care management among individuals living with hypertension. The key challenges identified included inadequate health literacy, low perceived susceptibility, limited instrumental and emotional support from family members and healthcare professionals, socially reinforced dietary norms that encourage excessive salt consumption, and uncertainty regarding the efficacy of treatment. Collectively, these barriers correspond to the principal constructs of the Theory: attitude, subjective norm, and perceived behavioural control, demonstrating how sociocultural contexts and habitual practices may shape individuals’ intentions and adherence behaviours. 

Therefore, healthcare professionals are encouraged to implement patient-centered strategies that recognize the dynamic interplay between social identity, familial relationships, and personal motivation. Future interventions should be theoretically grounded and socio-behaviourally tailored to community realities, particularly within rural and semi-urban Indonesian settings, to strengthen adherence and foster sustainable self-care behaviours.

## Supplementary Information

Supplementary Material 1.

Supplementary Material 2.

Supplementary Material 3.

## Data Availability

The datasets generated and analyzed during the current study are not publicly available to protect participant confidentiality, however, they are available from the corresponding author upon reasonable request.
