# Supplementary material for: Exploring the social and behavioral barriers to hypertension self-care among indonesian adults: a qualitative study based on the theory of planned behavior
Source: BMC Public Health. 2026 May 11;26:2045. doi: 10.1186/s12889-026-27470-6 (PMC13335283; doi:10.1186/s12889-026-27470-6)
Supplement: Supplementary file 1 — Supplementary Material 1. [file 12889_2026_27470_MOESM1_ESM.docx]

**Supplementary File 1: Semi-Structured Interview Guides (Indonesia Version)**

This supplementary file presents the semi-structured interview guides used in this qualitative study. The interview guides were developed based on the Theory of Planned Behavior (TPB) and adapted to the sociocultural context of the study setting. All interviews were conducted in Indonesian.

**Interview Guide for Family Members of Patients with Uncontrolled Hypertension**

| **No** | **Components** | **Description** |
| --- | --- | --- |
| 1 | Purpose | 1. To explore family members’ experiences and perceptions in supporting blood pressure control and salt preference management. 2. To identify barriers and facilitating factors influencing family support for hypertension self-care. 3. To identify strategies and interventions needed to support patients in controlling blood pressure and salt intake. |
| 2 | Opening | 1. Self-introduction 2. Explanation of study purpose and benefits 3. Estimated interview duration (approximately 60 minutes) 4. Assurance of confidentiality and use of participant codes |
| 3 | Key Questions | General Information:   1. Berapa usia anda? 2. Apa latar belakang Pendidikan dan pekerjaan anda?   Relationship with the patient   1. Apa hubungan anda dengan pasien? 2. Sudah berapa lama anda tinggal dan merawat pasien?   Attitude toward the behavior (attitude)   1. Bagaimana pandangan anda tentang pentingnya mengontrol tekanan darah anggota keluarga anda? 2. Menurut anda, apa manfaat dan kerugian dari pengurangan konsumsi garam pada pasien hipertensi?   Subjective norms   1. Bagaimana pengaruh keluarga dala keputusan pasien untuk mengontrol hipertensi? 2. Bagaimana pendapat orang-orang terdekat terhadap upaya anda mendukung pasien?   Perceived behavioral control   1. Seberapa mudah atau sulit anda membantu pasien mengurangi konsumsi garam dan mengontrol tekanan darah? 2. Kendala apa yang anda hadapi dan bagaimana cara mengatasinya?   Behavioral intention   1. Seberapa besar niat anda untuk terus mendukung pasien dalam mengontrol hipertensi? 2. Apa yang memotivasi anda untuk melakukan upaya tersebut secara berkelanjutan?   Intervention needs and recommendation   1. Apakah informasi dan dukungan yang anda terima sudah sesuai dengan kebutuhan anda? 2. Program atau bantuan apa yang dibutuhkan untuk mendukung pasien? |
| 4 | Closing Question | Apakah ada hal lain yang anda ingin sampaikan terkait penglaman dalam mendukung anggota keluarga anda yang hipertensi? |

**Interview Guide for Nurses**

| **No** | **Components** | **Description** |
| --- | --- | --- |
| 1 | Purpose | 1. To explore nurses’ experiences and roles in managing patients with hypertension. 2. To identify barriers and facilitating factors in hypertension management programs. 3. To identify strategies and interventions needed to improve hypertension care. |
| 2 | Opening | 1. Self-introduction 2. Explanation of study purpose and benefits 3. Estimated interview duration (approximately 60 minutes) 4. Cofidentiality assurance |
| 3 | Key Questions | Professional Background   1. Dimana anda bekerja dan apa tanggung jawab anda? 2. Sudah berapa lama anda memegang program hipertensi atau penyakit tidak menular (PTM)?   Implementation of Hypertension programs   1. Bagaimana pelaksanaan program hipertensi di puskesmas? 2. Seberapa sering kegiatan dilakukan setiap bulan?   Attitude toward the behavior (attitude)  Bagaimana peran dan pengalaman anda dalam pengelolaan hipertensi?  Subjective norms   1. Seberapa besar pengaruh kebijakan dan program yang ada terhadap pengelolaan hipertensi? 2. Faktor apa saja yang mempengaruhi pelaksanaan program?   Perceived behavioral control  Kendala apa yang anda hadapi dalam membantu pasien mengontrol tekanan dan dan asupan garam?  Behavioral intention   1. Seberapa besar niat anda untuk terus menjalankan program pengelolaan hipertensi? 2. Apa yang memotivasi anda untuk menjalankan program tersebut?   Intervention needs and recommendation  Strategi apa yang telah dan dapat dilakukan untuk meningkatkan efektivitas program? |
| 4 | Closing Question | Apakah ada hal lain yang anda ingin sampaikan terkait pengalaman dalam menjalankan program pengelolaan hipertensi |

**Interview Guide for Community Health Workers (PTM Cadres)**

| **No** | **Components** | **Description** |
| --- | --- | --- |
| 1 | Purpose | 1. To explore experiences and roles of community health workers in supporting hypertension care 2. To identify barriers and facilitating factors in community based hypertension management 3. To identify strategies to improve community hypertension self care support at the community level |
| 2 | Opening | 1. Self-introduction 2. Explanation of study purpose and benefits 3. Estimated interview duration (approximately 60 minutes) 4. Cofidentiality assurance |
| 3 | Key Questions | Professional Background   1. Apa latar belakang Pendidikan anda? 2. Apa peran anda sebagai kader? 3. Sudah berapa lama anda terlibat dalam program hipertensi atau penyakit tidak menular (PTM)?   Implementation of Hypertension programs   1. program pengelolaan hipertensi apa saja yang sudah berjalan? 2. Bagaimana peran anda dalam membantu puskesmas merawat pasien hipertensi?   Attitude toward the behavior (attitude)  Bagaimana peran dan pengalaman anda dalam membantu pengelolaan hipertensi?  Subjective norms   1. Seberapa besar pengaruh kebijakan dan program yang ada terhadap pengelolaan hipertensi yang anda lakukan? 2. Bagaimana dukungan dari puskesmas dan komunitas dalam kegiatan anda?   Perceived behavioral control  Kendala dan tantangan apa yang anda hadapi dalam membantu pasien mengontrol tekanan dan dan asupan garam?  Behavioral intention   1. Seberapa besar niat anda untuk terus menjalankan program pengelolaan hipertensi? 2. Apa yang memotivasi anda untuk menjalankan program tersebut?   Intervention needs and recommendation   1. Apakah kebutuhan utama untuk meningkatkan pengelolaan hipertensi di komunitas? 2. Apa rekomendasi anda agar program lebih efektif? |
| 4 | Closing Question | Apakah ada hal lain yang anda ingin sampaikan terkait pengalaman dalam menjalankan program pengelolaan hipertensi |

**Interview Guide for patients with uncontrolled hypertension**

| **No** | **Components** | **Description** |
| --- | --- | --- |
| 1 | Purpose | 1. To explore patients experiences and perceptions in controlling blood pressure and salt preference 2. To identify barriers and facilitating factors influencing hypertension self-care 3. To identify strategies and support needed for sustainable blood pressure control and salt reduction |
| 2 | Opening | 1. Self-introduction 2. Explanation of study purpose and benefits 3. Estimated interview duration (approximately 60 minutes) 4. Cofidentiality assurance |
| 3 | Key Questions | Professional Background   1. Berapa usia anda? 2. Apa latar belakang Pendidikan anda? 3. Sudah berapa lama anda menderita hipertensi   Attitude toward the behavior (attitude)   1. Apa yang anda ketahui tentang hipertensi? 2. Bagaimana pandangan anda tentang pentingnya mengontrol tekanan darah dan mengurangi asupan garam? 3. Menurut anda, apa manfaat dan kerugian dari mengurangi konsumsi garam?   Subjective norms   1. Seberapa besar pengaruh keluarga, tenaga kesehatan dan kader dalam keputusan anda mengontrol tekanan darah dan ambang rasa asin? 2. Bagaimana pendapat orang-orang disekitar anda tentang upaya anda dalam mengontrol tekanan darah?   Perceived behavioral control   1. Seberapa mudah atau sulit bagi anda untuk mengontrol tekanan darah dan mengurangi asupan garam? 2. Kendala apa saja yang anda hadapi dalam menjalankan diet, aktivitas fisik, minum obat, dan memantau tekanan darah?   Behavioral intention   1. Seberapa besar niat anda untuk terus mengontrol tekanan darah? 2. Apa yang memotivasi anda untuk melakukan pengelolaan tekanan darah dan pengurangan asupan garam secara berkelanjutan?   Intervention needs and recommendation   1. Dukungan atau informasi apa yang selama ini anda terima? 2. Bantuan atau program apa yang anda harapkan untuk membantu mengelola tekanan darah dan pengurangan asupan garam anda? |
| 4 | Closing Question | Apakah ada hal lain yang anda ingin sampaikan terkait pengalaman dalam mengontrol tekanan darah dan pengurangan asupan garam? |
