# Supplementary material for: Exploring the social and behavioral barriers to hypertension self-care among indonesian adults: a qualitative study based on the theory of planned behavior
Source: BMC Public Health. 2026 May 11;26:2045. doi: 10.1186/s12889-026-27470-6 (PMC13335283; doi:10.1186/s12889-026-27470-6)
Supplement: Supplementary file 2 — Supplementary Material 2. [file 12889_2026_27470_MOESM2_ESM.docx]

**Supplementary File 2: Expert panel of validation of the semi-structured interview guide**

**Purpose of Expert Validation**

This supplementary file describes the expert panel validation process undertaken to ensure the relevance, clarity, cultural appropriateness, and theoretical alignment of the semi-structured interview guide used in this qualitative study. The validation process aimed to enhance the methodological rigor and trustworthiness of the data collection instrument prior to formal data collection.

**Interview Guide Development**

A semi-structured interview guide was initially developed based on:

1. A literature review on hypertension self-care, salt reduction, and socio-behavioral determinants of chronic disease management, and
2. The theoretical framework of the Theory of Planned Behavior (TPB), which focuses on its three core constructs: attitudes, subjective norms, and perceived behavioral control.

Separate interview guides were prepared for each informant group (patients with uncontrolled hypertension, family members, community health workers/cadres, and nurses) to elicit role-specific experiences and perspectives regarding hypertension self-care.

**Composition of the Expert Panel**

The interview guide was reviewed by an interdisciplinary expert panel consisting of four experts with complementary expertise relevant to the study objectives:

1. A community nursing expert with experience in hypertension management and community-based care
2. A medical doctor with expertise in primary care and non-communicable disease management
3. A nutritionist with expertise in dietary behavior and salt intake
4. A medical anthropologist with expertise in sociocultural and behavioral aspects of health

All experts had prior experience in qualitative research and were familiar with the application of behavioral theories in health research.

**Validation Procedure**

Each expert independently reviewed the interview guide and evaluated the questions based on the following criteria:

1. **Relevance** to the study objectives
2. **Clarity** and comprehensibility of wording
3. **Cultural appropriateness** for the Indonesian context
4. **Conceptual alignment** with the TPB constructs

Experts provided written and verbal feedback on question wording, sequencing, and probing strategies. A consensus-based qualitative approach was used to determine whether items should be retained, revised, or clarified. No quantitative scoring system (e.g., Content Validity Index) was applied, consistent with established qualitative research practices emphasizing expert judgment and consensus.

**Revisions Following Expert Review**

Based on expert feedback:

1. Several questions were **reworded** to improve clarity and reduce ambiguity
2. Probing questions were **refined** to better capture social and family influences on self-care behaviors
3. Terminology related to dietary practices and salt consumption was **adapted** to enhance cultural relevance

No interview questions were removed. All revisions were incorporated through team discussion until agreement was reached among the research team.

### ****Pilot Testing****

Following expert validation, the revised interview guide was pilot-tested with five individuals with hypertension from a community outside the study setting. Feedback from pilot participants indicated that the questions were understandable and culturally appropriate. Minor adjustments were made to improve flow and sequencing prior to final data collection.

### ****Final Interview Guide****

The finalized semi-structured interview guide used for data collection is provided in **Supplementary File 1 (Indonesian version).**

### ****Methodological Rigor****

The expert panel validation process strengthened the credibility and dependability of the study by ensuring that the interview guide was theoretically grounded, culturally sensitive, and appropriate for exploring social-behavioral barriers to hypertension self-care across multiple informant groups.

**Appendix: Expert assessment form for the semi-structured interview guide**

### ****Study Title****

A Qualitative Study of Social-Behavioral Barriers to Hypertension Self-Care in Indonesia: Extending the Theory of Planned Behavior

**Expert information**

Name :

Area of expertise :

Professional role :

## **Instructions for Experts**

Please review the attached semi-structured interview guide and provide qualitative feedback on each assessment domain below.
Your comments will be used to improve the clarity, relevance, cultural appropriateness, and theoretical alignment of the interview questions.
There are **no right or wrong answers**. Please focus on **constructive suggestions**.

**Section A: Expert Assessment form**

| **Assessment Domain** | **Guiding Question** | **Expert Judgment** | **Comment/Suggestions** |
| --- | --- | --- | --- |
| Relevance to study objective | Do the interview questions adequately capture social-behavioral barriers to hypertension self-care? | ☐ Very relevant  ☐ Relevant  ☐ Needs revision  ☐ Not relevant |  |
| Conceptual alignment with TPB | Are the questions aligned with TPB constructs (attitude, subjective norm, perceived behavioral control)? | ☐ Well aligned  ☐ Adequately aligned  ☐ Partially aligned  ☐ Not aligned |  |
| Clarity and comprehensibility | Are the questions clearly worded and understandable for participants with diverse educational backgrounds? | ☐ Very clear  ☐ Clear  ☐ Some questions unclear  ☐ Many questions unclear |  |
| Cultural appropriateness | Are the questions culturally appropriate for the Indonesian context (family roles, communal practices)? | ☐ Highly appropriate ☐ Appropriate  ☐ Needs cultural adjustment  ☐ Not appropriate |  |
| Appropriateness across informant groups | Are the questions suitable for patients, family members, cadres, and nurses? | ☐ Very appropriate ☐ Appropriate  ☐ Some adjustment needed  ☐ Major adjustment needed |  |
| Sequencing and flow | Is the order of questions logical and conducive to in-depth discussion? | ☐ Very good  ☐ Good  ☐ Needs minor revision  ☐ Needs major revision |  |
| Probing questions | Are the probes sufficient to elicit rich and meaningful data? | ☐ Very sufficient  ☐ Sufficient  ☐ Needs additional probes  ☐ Insufficient |  |

**Section B: Overall Feedback**

| **Aspect** | **Expert Comments** |
| --- | --- |
| \| Key strengths of the interview guide \|  \| \| --- \| --- \| |  |
| \| Areas requiring improvement \|  \| \| --- \| --- \| |  |
| \| Suggestions for additional or revised questions \|  \| \| --- \| --- \| |  |
| \| Overall assessment of the interview guide \| \| --- \| |  |

**Expert Declaration**

|  |
| --- |
|  |
|  |
|  |

**Expert Signature**

| **Name** | **Signature** | **Date** |
| --- | --- | --- |
|  |  |  |
