# Supplementary material for: Exploring the social and behavioral barriers to hypertension self-care among indonesian adults: a qualitative study based on the theory of planned behavior
Source: BMC Public Health. 2026 May 11;26:2045. doi: 10.1186/s12889-026-27470-6 (PMC13335283; doi:10.1186/s12889-026-27470-6)
Supplement: Supplementary file 3 — Supplementary Material 3. [file 12889_2026_27470_MOESM3_ESM.docx]

**Supplementary File 1: Semi-Structured Interview Guides (English Version)**

This supplementary file presents the semi-structured interview guides used in this qualitative study. The interview guides were developed based on the Theory of Planned Behavior (TPB) and adapted to the sociocultural context of the study setting. The questions were designed to explore participants’ attitudes, subjective norms, perceived behavioral control, and behavioral intentions related to hypertension self-care and salt preference. All interviews were originally conducted in Indonesian. The following is the English version of the interview guide used for analysis and reporting.

**Interview Guide for Family Members of Patients with Uncontrolled Hypertension**

| **No** | **Components** | **Description** |
| --- | --- | --- |
| 1 | Purpose | 1. To explore family members’ experiences and perceptions in supporting blood pressure control and salt preference management. 2. To identify barriers and facilitating factors influencing family support for hypertension self-care. 3. To identify strategies and interventions needed to support patients in controlling blood pressure and salt intake. |
| 2 | Opening | 1. Self-introduction 2. Explanation of study purpose and benefits 3. Estimated interview duration (approximately 60 minutes) 4. Assurance of confidentiality and use of participant codes |
| 3 | Key Questions | General Information:   1. What is your age? 2. What is your educational background and occupation?   Relationship with the patient   1. What is your relationship with the patient? 2. How long have you been living with or caring for the patient?   Attitude toward the behavior (attitude)   1. What are your views on the importance of controlling blood pressure? 2. What do you think are the benefits and disadvantages of reducing salt intake?   Subjective norms   1. How does family influence the patient’s decision to manage hypertension? 2. How do people around you perceive your perceive your efforts to support the patient?   Perceived behavioral control   1. How easy or difficult is it to help the patient reduce salt intake and control blood pressure? 2. What challenges do you face and how do you overcome them?   Behavioral intention   1. How strong is your intention to continue supporting the patient? 2. What motivates you to do so?   Intervention needs and recommendation   1. What kind of support or information do you need? 2. What types of programs would help you better support the patient? |
| 4 | Closing Question | Is there anything else you would like to share? |

**Interview Guide for Nurses**

| **No** | **Components** | **Description** |
| --- | --- | --- |
| 1 | Purpose | 1. To explore nurses’ experiences and roles in managing patients with hypertension. 2. To identify barriers and facilitating factors in hypertension management programs. 3. To identify strategies and interventions needed to improve hypertension care. |
| 2 | Opening | 1. Self-introduction 2. Explanation of study purpose and benefits 3. Estimated interview duration (approximately 60 minutes) 4. Cofidentiality assurance |
| 3 | Key Questions | Professional Background   1. Where do you currently work, and what are your main responsibilities? 2. How long have you been involved in managing hypertension or non-communicable disease (NCD) programs?   Implementation of Hypertension programs   1. How are hypertension management programs implemented at your primary healthcare center? 2. How frequently are these program activities conducted each month?   Attitude toward the behavior (attitude)  How would you describe your role and experience in managing hypertension care?  Subjective norms   1. To what extent do existing policies and programs influence hypertension management practices? 2. What factors affect the implementation of these programs?   Perceived behavioral control  What challenges do you face in helping patients control their blood pressure and reduce salt intake?  Behavioral intention   1. How strong is your intention to continue implementing hypertension management programs? 2. What motivates you to sustain your involvement in these programs?   Intervention needs and recommendation  What strategies have been implemented, or could be implemented, to improve the effectiveness of hypertension management programs? |
| 4 | Closing Question | Is there anything else you would like to share regarding your experience in implementing hypertension management programs? |

**Interview Guide for Community Health Workers**

| **No** | **Components** | **Description** |
| --- | --- | --- |
| 1 | Purpose | 1. To explore experiences and roles of community health workers in supporting hypertension care 2. To identify barriers and facilitating factors in community based hypertension management 3. To identify strategies to improve community hypertension self-care support at the community level |
| 2 | Opening | 1. Self-introduction 2. Explanation of study purpose and benefits 3. Estimated interview duration (approximately 60 minutes) 4. Cofidentiality assurance |
| 3 | Key Questions | Professional Background   1. What is your educational background? 2. What is your role as a community health worker? 3. How long have you been involved in hypertension or non-communicable disease (NCD) programs   Implementation of Hypertension programs   1. What hypertension management programs are currently being implemented in your community? 2. What is your role in supporting primary healthcare centers in managing patients with hypertension?   Attitude toward the behavior (attitude)  How would you describe your role and experience in supporting hypertension management?  Subjective norms   1. To what extent do existing policies and programs influence your activities in hypertension management? 2. How would you describe the support you receive from primary healthcare centers and the community in carrying out your activities?   Perceived behavioral control  What challenges and barriers do you encounter in helping patients control their blood pressure and reduce salt intake?  Behavioral intention   1. How strong is your intention to continue participating in hypertension management programs? 2. What motivates you to remain involved in these programs?   Intervention needs and recommendation   1. What are the main needs to improve hypertension management at the community level? 2. What recommendations would you suggest to enhance the effectiveness of these programs? |
| 4 | Closing Question | Is there anything else you would like to share regarding your experience in supporting hypertension management? |

**Interview Guide for Patients with Uncontrolled Hypertension**

| **No** | **Components** | **Description** |
| --- | --- | --- |
| 1 | Purpose | 1. To explore patients experiences and perceptions in controlling blood pressure and salt preference 2. To identify barriers and facilitating factors influencing hypertension self-care 3. To identify strategies and support needed for sustainable blood pressure control and salt reduction |
| 2 | Opening | 1. Self-introduction 2. Explanation of study purpose and benefits 3. Estimated interview duration (approximately 60 minutes) 4. Cofidentiality assurance |
| 3 | Key Questions | Professional Background   1. What is your age? 2. What is your educational background? 3. How long have you been diagnosed with hypertension?   Attitude toward the behavior (attitude)   1. Can you describe your understanding of hypertension? 2. How do you perceive the importance of controlling blood pressure and reducing salt intake? 3. In your opinion, what are the benefits and disadvantages of reducing salt consumption?   Subjective norms   1. To what extent do family members, healthcare providers, and community health workers influence your decisions to control blood pressure and manage salt intake? 2. How do people around you perceive your efforts to control your blood pressure?   Perceived behavioral control   1. How easy or difficult is it for you to control your blood pressure and reduce salt intake? 2. What challenges do you face in maintaining a healthy diet, engaging in physical activity, taking medication, and monitoring your blood pressure?   Behavioral intention   1. How strong is your intention to continue managing your blood pressure over time? 2. What motivates you to sustain blood pressure control and reduce salt intake over time?   Intervention needs and recommendation   1. What kind of support or information have you received so far? 2. What types of support or programs would help you better manage your blood pressure and reduce salt intake? |
| 4 | Closing Question | Is there anything else you would like to share regarding your experience in controlling your blood pressure and reducing salt intake? |
